# Intestinal helminths as predictors of some malaria clinical outcomes and IL-1β levels in outpatients attending two public hospitals in Bamenda, North West Cameroon

**DOI:** 10.1371/journal.pntd.0009174

**Published:** 2021-03-02

**Authors:** Helen Ngum Ntonifor, Julius Suh Chewa, Mahamat Oumar, Hermann Desire Mbouobda

**Affiliations:** 1 Department of Biological Sciences, Faculty of Science, University of Bamenda, Bambili, North West Region, Cameroon; 2 Department of Biology, Higher Teachers Training College, University of Bamenda, Bambili, North West Region, Cameroon; NIH-National Institute for Research in Tuberculosis-ICER, INDIA

## Abstract

This study aimed at determining the impact of intestinal helminths on malaria parasitaemia, anaemia and pyrexia considering the levels of IL-1β among outpatients in Bamenda. A cohort of 358 consented participants aged three (3) years and above, both males and females on malaria consultation were recruited in the study. At enrolment, patients’ axillary body temperatures were measured and recorded. Venous blood was collected for haemoglobin concentration and malaria parasitaemia determination. Blood plasma was used to measure human IL-1β levels using Human ELISA Kit. The Kato-Katz technique was used to process stool samples. Five species of intestinal helminths *Ascaris lumbricoides* (6.4%), *Enterobius vermicularis* (5.0%), *Taenia* species (4.2%), *Trichuris trichiura* (1.1%) and hookworms (0.8%) were identified. The overall prevalence of *Plasmodium falciparum* and intestinal helminths was 30.4% (109/358) and 17.6% (63/358) respectively. The prevalence of intestinal helminths in malaria patients was 17.4% (19/109). Higher Geometric mean parasite density (GMPD ±SD) (malaria parasitaemia) was significantly observed in patients co-infected with *Enterobius vermicularis* (5548 ± 2829/μL, *p* = 0.041) and with *Taenia* species (6799 ± 4584/μL, *p* = 0.020) than in *Plasmodium falciparum* infected patients alone (651 ± 6076/ μL). Higher parasitaemia of (1393 ± 3031/μL) and (3464 ± 2828/μL) were recorded in patients co-infected with *Ascaris lumbricoides* and with hookworms respectively but the differences were not significant (p > 0.05). Anaemia and pyrexia prevalence was 27.1% (97/358) and 33.5% (120/358) respectively. Malaria patients co-infected with *Enterobius vermicularis* and *Ascaris lumbricoides* had increased risk of anaemia (OR = 13.712, *p =* 0.002 and OR = 16.969, *p* = 0.014) respectively and pyrexia (OR = 18.07, *p* = 0.001 and OR = 22.560, *p* = 0.007) respectively than their counterparts. Increased levels of IL-1β were significantly observed in anaemic (148.884 ± 36.073 pg/mL, t = 7.411, *p* = 0.000) and pyretic (127.737 ± 50.322 pg/mL, t = 5.028, *p* = 0.000) patients than in non-anaemic (64.335 ± 38.995pg/mL) and apyretic patients (58.479 ± 36.194pg/mL). Malaria patients co-infected with each species of intestinal helminths recorded higher IL-1β levels (IL-1β > 121.68 **±** 58.86 pg/mL) and the overall mean (139.63 **±** 38.33pg/mL) was higher compared with levels in malaria (121.68 **±** 58.86 pg/mL) and helminth (61.78 **±** 31.69pg/mL) infected patients alone. Intestinal helminths exacerbated the clinical outcomes of malaria in the patients and increased levels of IL-1β were observed in co-infected patients with anaemia, pyrexia and higher parasitaemia.

## Author summary

Malaria and intestinal helminthiasis are endemic parasitic diseases in Sub Sahara Africa including Cameroon that has been associated with poverty. Humans are co-infected with these diseases. Intestinal helminths have been reported to improve or exacerbate malaria severities in co-infected patients. The precise mechanism through which they exert this is not well elucidated but there are speculations about possible immunological implications. It is therefore crucial to understand the mechanism how these neglected tropical disease (helminthiasis) impact malaria severities to develop robust integrated public health intervention strategies and treatment protocols that can effectively manage these diseases in endemic zones. In this study, we focused on the impact of these helminths on malaria parasitaemia, anaemia and pyrexia. We examined each patient for malaria and helminth infections while also measuring their haemoglobin concentrations and body temperatures. We found out that patients infected with intestinal helminths had increased risk of malaria infection and exacerbated malaria parasitaemia, anaemia and pyrexia in co-infected patients. We also observed that increased levels of IL-1β were higher in these co-infected patients than in patients infected with malaria parasite or helminths alone. Our study is informative about the possible involvement of intestinal helminths with the immune responses of the host that consequently affects malaria severity.

## Introduction

Malaria is an endemic disease in Sub-Saharan Africa. A protozoan called *Plasmodium* causes it and *Plasmodium falciparum* remains the most prevalent species in this region [1]. *Plasmodium falciparum* infects about 93% of Sub Saharan Africans recording a mortality rate of 94% out of the global cases and deaths [2]. The prevalence of malaria in Cameroon has been reported at 30.3% with over one (1) million cases and about ten (10) thousand deaths reported annually in the country’s health facilities [3]. Added to malaria, about 24% of the world’s population (1.5 billion people) are infected with helminths [4] with about ten (10) million Cameroonians infected [5]. Intestinal helminths such as *Ascaris lumbricoides*, *Trichuris trichiura*, hookworms (*Ancylostoma duodenale* and *Necator americanus*), *Enterobius vermicularis* and *Taenia* species (*Taenia solium* and *Taenia saginata*) are also prevalent in Sub Sahara Africa [4,6,7,8] and co-infect malaria patients in endemic zones including Cameroon [9,10,11] with a co-infection prevalence of 11.6% amongst children [12]. The thriving of these parasitic worms in the tropical regions is associated to poverty, poor sanitation and hygiene, and poor health awareness. The effect of their presence on the clinical outcomes and other malaria severities remains contradictory.

Some studies have reported protective effect of intestinal helminths on malaria severities [10,13,14,15], while others have reported that intestinal helminths have deleterious effect on malaria severities [16,17]. For example, some studies have reported *Ascaris lumbricoides* to be associated with a reduction of malaria parasite density [14,15] and protection against severe malaria complications [13] and anaemia in children [10]. While in other studies, it was associated with increased malaria severity in children [16]. Recent studies have reported severe anaemia amongst malaria patients co-infected with intestinal helminths [11,18,19]. In Cameroon, studies seeking to investigate the effect of intestinal helminths on the clinical outcomes of malaria [10,12] have not specifically focus on the effect of coinfection on pyrexia. Meanwhile, the impact of these helminths on malaria parasitaemia and anaemia are still conflicting and the mechanism underlying the impact of intestinal helminths on malaria severity still remain unknown.

There are speculations that intestinal helminths could possibly have some immunological impact on malaria severities. However, these claims are still under investigations. The understanding of the association between inflammatory cytokines and malaria severities is complex. Human immune responses against malaria parasites is characterized by marked changes in the level of proinflammatory cytokines such as IL-8, IL-10, IL-12 [20]. These mediators alongside endogenous pyrogens like interleukin IL-1β, IL-6 and Tumor necrosis factor alpha (TNF-α) have been associated with malarial pyrexia [21] and other complications. Increased levels of IL-10 [22] and TNF-α [23] have been implicated with high and low malaria parasitaemia amongst humans respectively. In a study conducted among human subjects on inflammatory cytokines in *falciparum* malaria [20], mild level of IL-8 and significantly elevated levels of IL-6, IL-10, IL-12 and TNF-α correlated with severe malaria. The study also recorded that lower levels of IL-6 and IL-10 were noted in subjects with severe anaemia and specifically, low levels of IL-6 were associated with higher malaria parasitaemia in the subjects. However, there are contradictory information about the effect of IL-1β on malaria severities [20,24,25] and the study did not situate the level of IL-1β with any malaria severity and suggest a possible downregulation of IL-1β by IL-10 [20]. Intestinal helminths may enhance their establishing within the host through the inducement of type II (T_2_H) immune responses that involves the production of the anti-inflammatory cytokine IL-10 [26]. These worms modulate host immune responses both to themselves and other infecting parasite [27]. In Cameroon some studies have sought to investigate the possible immunological implications of helminths on malaria severities but the studies have been limited to immunoglobulins [28]. One of the objectives of this study was to assess the plasma levels of IL-1β in a cross section of outpatients that were infected or coinfected with malaria parasite and intestinal helminths, and further associate the plasma IL-1β levels with anaemia, pyrexia and malaria parasitaemia. In this study we hypothesized that intestinal helminth coinfections with *Plasmodium falciparum* and IL-1β levels are associated with the clinical outcomes of malaria. Therefore, this study aimed at assessing the implications of intestinal helminths on malaria clinical outcomes focusing on trends in malaria parasitaemia, anaemia and pyrexia among outpatients coinfected with *Plasmoidum falciparum* and intestinal helminths, with emphasis on the variation in plasma IL-1β levels among patients with different clinical and parasitological status.

## Materials and methods

### Ethics statement

This study was approved by the Institutional Review Board (IRB) of the Regional Delegation of Public Health for the North West Region (No.072/APP/RDPH/RHB/IRB). An authorization was also obtained from the Regional Delegation of Public Health for the North West Region (No.194/ATT/NWR/RDPH) and the different hospitals. The purpose and aims of the study was fully explained to the participants through an information sheet written in English language. For participants who could not read nor understand English language, the information was read and translated into “Pidgin English” for better comprehension. All blood and stool samples were collected after the subjects had provided their signed written informed consents and parents/guardians of minors (subject ≤ 18 years) signed the written informed consent for minors authorising them to take part in the study. No incentives were given to participants. Results of each participant were made known to them, and positive subjects were referred to a medical doctor for proper management and treatment. Participants were enlightened on the need to receive preventive treatments from the hospitals and on how to protect themselves from intestinal helminths and malaria.

### Study area

This study was conducted in the Regional Hospital Bamenda and the Medicalized Health Centre (CMA) Nkwen-Bamenda. Bamenda is the fifth largest city in Cameroon having a cosmopolitan population of about 393, 835 inhabitants distributed on a surface area of about 22.9 square kilometres. The geography of the city is characterised by cold, tropical climatic condition with a longer rainy season usually between late March to early November and shorter dry season between early November to late March. The city is located on longitude 5^0^56’ N and latitude 10^0^10’E at an altitude of 1,614 meters above sea level. Bamenda has an annual rainfall of about 2,145mm, annual temperature range of about 16°C– 25°C and mean monthly humidity of about 91%.

### Study design, study population and selection criteria

This study was a hospital based, observational study conducted in a cross section of outpatients between August 2019 to March 2020. Only outpatients aged three (3) years and above of both sexes who were on malaria consultation in two public hospitals in Bamenda were recruited. The population sample size was estimated from the proportion of malaria prevalence (30.3%) in Cameroon using the Cochran formula [29] as computed below. The calculated population sample size was adjusted by 11%. Only consented participants that showed an episode of fever within 48 hours took part in the study. Inpatients, HIV patients, pregnant women, patients with haemoglobinopathies, those already on anti-malarial and anthelminthic medications including those that had taken any of the medications for the past two months were all excluded from the study.

$$Sample\;size=z^{2}pq/e^{2}$$

Malariaprevalence(p)=0.3,q=1–p(0.7),z-score=1.96,errormargin(e)=0.05

$$Sample\;size={\left(1.96\right)}^{2}(0.3)(0.7)/{\left(0.05\right)}^{2}$$

Samplesize∼358participants

### Assessment of pyrexia

Axillary body temperature of each participant was measured using a clinical thermometer and values were recorded in degree Celsius. Patients with axillary body temperatures > 37.5°C were set to be pyretic and those with temperatures ≤ 37.5°C were defined as apyretic [21,30].

### Collection of blood samples

Venous blood (2–5 mLs) were collected from each recruited patient into labelled Ethylene diamine tetraacetate (EDTA) test tubes using a vacutainer. Blood samples were used for the detection of malaria parasite density, haemoglobin concentration and plasma IL-1β levels.

### Malaria parasite determination

Thin and thick blood films were made on clean labelled glass slides. The blood smears were dried using a hair dryer [31] and the thin films fixed using MayGrunwald stain for10 seconds. Both the thin and thick films were later stained with 10% Giemsa for 10 minutes [32]. The slides were carefully washed, dried and observed under the x100 (oil immersion) objective of a compound microscope (Olympus CX22, Olympus Corporation, Tokyo, Japan). The World Health Organization (WHO) bench aid for the diagnosis of malaria parasite [33] was used to identify any of the malaria parasites. Thin films were used for the identification of the malaria parasite species and the thick films used to quantify the malaria parasite density per microliter (μL) of blood. This was done by counting the asexual stage (trophozoites) against 200 leukocytes assuming total White Blood Cell (WBC) count of 8 000 leukocytes/ μl of blood [32]. Slides with no asexual or sexual stages of malaria parasite were reported as negative after observing up to 100 high power fields. Parasite densities were further classified as mild (MP < 1 000 parasites/μL), moderate (MP 1 000–4 999 parasites/μL) and severe (MP ≥ 5 000 parasites/μL) [34,35].

### Assessment of haemoglobin concentration

The URIT-12 haemoglobin meter was used to measure patient’s Haemoglobin (Hb) concentration in grams per decilitre of blood (g/dL). Immediately after collection of venous blood from each patient into labelled EDTA test tube, a drop of the blood was pipetted onto the URIT test strip already inserted in the machine and the value displayed in few seconds was recorded. This was done for all the participants and in compliance with the protocol of the manufacturer (URIT Medical electronic company, London, UK). Anaemia was defined by Hb < 11 g/dL of blood [36] and was further classified as mild (Hb 10–10.9 g/dL), moderate (Hb 7–9.9 g/dL) and severe (Hb < 7 g/dL) [32].

### Assessments of plasma IL-1β levels

The remaining parts of the blood samples in the EDTA test tubes were centrifuged at 3 000 revolutions per minute (rpm) for 15 minutes (Horizon centrifuge, Drunker diagnostics, USA) and the plasma pipetted into labelled Eppendorf tubes and stored at -20 ^0^C prior to immunological analysis. Human plasma IL-1β levels were analysed using Human IL-1β ELISA kit following the protocol (S1 Text) of the manufacturer (RayBiotech, Parkway Lane, GA, USA).

### Stool samples and intestinal helminths determination

Five grams of stool were collected from each participant into labelled sterile stool bottles. They were collected void of urine contamination. Collected stool samples were processed using the Kato-Katz technique following the protocol of the manufacturer (Kato-Katz kit, Sterlitech Corporation, Kent, WA, USA) to diagnose intestinal helminths [37]. The slides were read within sixty minutes to avoid missing eggs of hookworms and other helminths that may disintegrate during longer clearing time. The egg burden for each species of intestinal helminths were calculated as egg per gram (epg) of stool by multiplying total eggs of each helminth species on the slide by 24 following recommended stool templates of 41.7mg [38]. Slides that did not have any helminth eggs or larvae were reported as negative. The egg burden of the intestinal helminths were further classified as light, moderate or heavy infections [39,40]. Only single independent stool sample was collected from each participant and examined for the presence of intestinal helminths without post treatment diagnosis or assessment of the patients.

### Data analysis

Collected data were imputed into Microsoft Excel spreadsheet 2016 (Microsoft office, USA), cleaned, filtered, coded and uploaded into the Statistical Package for Social Sciences (SPSS) software version 23 (IBM SPSS Inc.Chicago, IL, USA) for statistical analysis. Mean differences between groups for normally distributed variables were assessed using the student t-test and One Way ANOVA (Analysis of Variance). Multiple comparisons within groups were computed using the Tukey Multi comparison Test. The binary logistic regression was used to assess level of association between variables. The malaria parasite density and helminth egg counts in co-infected samples were log transformed in base 10. The cut off point for assessing all statistical significance between groups was set at probability level (*p*) < 0.05.

## Results

### Baseline characteristics of the study population

A total of 358 outpatients of both sexes (Males 133 and females 225) that had experienced an episode of fever within 48 hours and were on malaria consultation in the public hospitals were recruited. The mean age (range) in the study was 31 ± 20 (3–62 years). At enrolment, the patients were examined for pyrexia and anaemia and 33.5% of them were pyretic while 24.3% were anaemic. The prevalence of mild, moderate and severe anaemia were 58.6%, 36.8% and 4.6% respectively.

### Prevalence and density of various parasite species among the study population

*Plasmodium falciparum* was the only *Plasmodium* species identified and was observed to infect 30.4% of the population registering a geometric mean parasite density (GMPD) of 831 ± 5764/μl of blood. Most patients (60.6%) had mild (MP < 1000 parasite/μL) malaria. Five species of intestinal helminths: *Ascaris lumbricoides*, *Enterobius vermicularis*, *Taenia* species, *Trichuris trichiura* and hookworms were identified and found to infect 17.6% of the population. *Ascaris lumbricoides* recorded the highest (6.4%) prevalence and least by hookworms (0.8%). Conversely, hookworms registered the highest geometric mean egg density (GMED) per gram of stool and least by *Trichuris trichiura* as shown in Table 1. With the exception of *Ascaris lumbricoides*, all intestinal helminth infections were of light infection. One patient was moderately (5 000–49 999 epg) infected with *Ascaris lumbricoides*.

**Table 1:** Prevalence, parasite density categories and geometric mean (GM ±Standard deviation) parasite load in the population.

Parasite species	Number examined	Number infected (%)	Parasite density category			GMPD (Range)
			Mild/light† n (%)	Moderate ‡ n (%)	Severe†† n (%)	
*P*. *falciparum*	358	109 (30.4)	66 (60.6)	25(22.9)	18(16.5)	831 (80–40400)
*A*. *lumbricoides*		23 (6.4)	22 (95.7)	1 (4.3)	0 (0)	133 (24–9072)
*T*. *trichiura*		4 (1.1)	4 (100)	0 (0)	0 (0)	50 (24–72)
Hookworms		3 (0.8)	3 (100)	0 (0)	0 (0)	144 (96–216)
*E*. *vermicularis*		18 (5.0)	18 (100)	0 (0)	0 (0)	89 (24–624)
*Taenia* species		15 (4.2)	15 (100)	0 (0)	0 (0)	51 (24–168)
Overall Helminth infection		63 (17.6)	62 (98.4)	1 (1.6)	0 (0)	-

a n indicate number of infected patients; % (prevalence)

† Mild: *P*. *falciparum* (< 1 000 parasite/ μl of blood); light: *Ascaris* (1–4 999 epg); *Trichuris* (1–999 epg); Hookworm (1–1 999epg).

‡ Moderate: *P*. *falciparum* (1 000–4 999 parasite/μl of blood); *Ascaris* (5 000–49 999 epg)

†† Severe: *P*. *falciparum* (≥ 5 000 parasite/ μl of blood)

GMPD: indicate geometric mean parasite density

### Prevalence of *Plasmodium falciparum* and intestinal helminth co-infections among outpatients

Out of 109 malaria patients recorded, nineteen (19) were co-infected with one or two species of intestinal helminths registering a co-infection morbidity rate of 17.4%. The *Plasmodium*/Helminth co-infection categories recorded in this study were *Plasmodium* /*Ascaris (Pf/Al)* 6.4% (7/109), *Plasmodium*/Hookworms (*Pf*/Hw) 1.8% (2/109), *Plasmodium/Enterobius* (*Pf/Ev*) 3.7% (4/109), *Plasmodium*/*Taenia* species (*Pf/Ts*) 3.7% (4/109), *Plasmodium/Ascaris/Trichuris* (*Pf/Al/Tt*) 0.9% (1/109), and *Plasmodium/Ascaris/Taenia species* (*Pf/Al/Ts*) 0.9% (1/109).

### The risk of intestinal helminth exposure to malaria infection

The logistic binary regression was used to evaluate the relationship between intestinal helminth infections and malaria after controlling the confounding effect of other intestinal helminth species. The odd ratios obtained from the logistic binary regression analysis were used to evaluate the risk of intestinal helminths to malaria. We observed generally that malaria patients infected with one or more species of intestinal helminths recorded higher odds (OR > 1) of *Plasmodium falciparum* infection except *Enterobius vermicularis* (OR = 0.861) but the differences were not significant (*p* > 0.05) as shown in Table 2.

**Table 2:** The risk of helminth infection to *falciparum* malaria.

Helminth species	Outcome: *falciparum* malaria		
	Odd Ratio	95% C.I^a^	*p–*value
*A*. *lumbricoides*	1.841	0.665–5.095	0.267
Hookworms	4.733	0.424–52.863	0.216
*E*. *vermicularis*	0.861	0.267–2.775	1.000
*Taenia* species	1.183	0.348–4.029	0.756
*Ascaris /Trichuris*	2.367	0.146–38.253	0.508
*Ascaris* /*Taenia* spp	1.183	0.106–13.216	1.000
*T*. *trichuris*	ND	ND	ND

a C.I indicate Confidence interval

ND: Not determined because of low count

### Malaria parasitaemia and helminth egg burden in patients with co-infection

The parasite densities were log transformed in base 10 and intestinal helminths had a high significant impact (F = 5.007, *p =* 0.001) on the mean malaria parasite (parasitaemia) density (MMPD) in co-infected patients. Significantly higher MMPDs were recorded in patients co-infected with *Pf/Ts* (MMPD: 3.83 ± 0.27, *p =* 0.020) and *Pf/Ev* (MMPD: 3.74 ± 0.19, *p =* 0.041) than in patients infected with *Plasmodium falciparum* alone (2.81 ± 0.66) as shown in Table 3. We also, observed an insignificantly heightened MMPD in patients co-infected with *Ascaris lumbricoides* (MMPD: 3.14 ± 0.69, *p =* 0.681) and hookworms (MMPD: 3.54 ± 0.34, *p =* 0.508). There were no significant differences (*p* > 0.05) in the mean egg densities (MED) between helminth-infected patients and co-infected patients.

**Table 3:** Comparison of *Plasmodium falciparum* and helminth mean densities between co-infected and non co-infected patients.

Parasite status	Cases	MMPD ± SD†	*p–value*	MED ± SD‡	*p—value*
*Pf* only	90	2.81 ± 0.66	Reference	-	-
*Pf /Al*	7	3.14 ± 0.69	0.681	2.13 ± 0.49	0.999
*Pf /Hw*	2	3.54 ± 0.34	0.508	2.25 ± 0.12	-
*Pf /Ev*	4	3.74 ± 0.19	0.041	1.90 ± 0.41	0.999
*Pf* /Ts	4	3.83 ± 0.27	0.020	1.86 ± 0.26	0.962
*Al* only	9	**-**	**-**	2.09 ± 0.35	Reference
*Ev* only	11	**-**	**-**	1.99 ± 0.54	Reference
Ts only	8	**-**	**-**	1.66 ± 0.24	Reference
Significant difference		F = 5.007 *p =* 0.001		F = 1.568 *p =* 0.192	
*Tt* only	1	ND	ND	1.85 ± 0.00	ND
Hw only	1	ND	ND	1.98 ± 0.00	ND
*Pf/Al/Tt*	1	2.72 ± 0.00	ND	Al: 1.68 ± 0.00 Tt: 1.38 ± 0.00	ND ND
*Pf/Al/*Ts	1	2.89 ± 0.00	ND	Al: 1.86 ± 0.00 Ts: 1.38 ± 0.00	ND ND

***Note***: The parasite densities were log transformed to base 10

† MMPD indicate Mean Malaria Parasite Density.

‡ MED indicate Mean Egg Density.

ND: Not determine because the cases were exactly one.

### Effect of intestinal helminths on haemoglobin concentrations and temperatures in malaria patients

We observed a significant (*p <* 0.0001) change in the mean haemoglobin (Hb) concentrations in patients infected with malaria parasite and in those co-infected with intestinal helminths. Patients that were infected with *Plasmodium falciparum* alone were mildly anaemic (Hb 10.9 ± 1.99 g/dL) and had significantly (*p =* 0.001) low mean haemoglobin concentrations than participants that were not infected with any parasite at all (12.6 ± 1.66 g/dL). In addition, malaria patients co-infected with hookworms or *E*. *vermicularis* excluding *Ascaris* and *Taenia* species were also mildly anaemic though recorded insignificant (*p >* 0.05) lower mean haemoglobin concentrations of 10.0 ± 0.42 g/dL and 10.4 ± 0.86 g/dL respectiely compared with patients infected with *Plasmodium falciparum* alone (Hb 10.9 ± 1.99 g/dL). Also, malaria patients co-infected with double species of intestinal helminths were moderately anaemic (Hb < 10 g/dL) and had lower haemoglobin concentration levels than patients infected with *Plasmodium falciparum* alone and malaria patients co-infected with single species of helminths as shown in Table 4. We also noticed that patients infected alone with *Ascaris lumbricoides*, *Enterobius vermicularis* and *Taenia* species were not anaemic (Hb ≥ 10 g/dL) and had elevated mean haemoglobin concentration levels than their counterparts co-infected with *Plasmodium falciparum*.

**Table 4:** Mean haemoglobin concentration levels and temperature in patients with different parasitic infections and coinfections.

Parasite status	Case	Hb concentration(g/dL)		*p*—value	Temperature (^0^C)		*p*—value
		Mean ± SD	Range		Mean ± SD	Range	
*Pf* only	90	10.9 ± 1.99	3.6–15.2	< 0.001	37.9 ± 0.6^a^	36.1–39.8	< 0.001*
*Pf /Al*	7	11.4 ± 2.16	10.3–16.3	> 0.05	37.9 ± 0.7	37.4–39.3	< 0.01*, > 0.05^a^
*Pf /Hw*	2	10.0 ± 0.42	9.7–10.3		37.9 ± 0.0	37.9–37.9	> 0.05*^a^
*Pf /Ev*	4	10.4 ± 0.86	9.7–11.6		38.1 ± 1.1	36.9–39.5	< 0.01*, > 0.05^a^
*Pf* /Ts	4	11.6 ± 1.76	9.9–13.4		38.6 ± 0.6	37.9–39.4	< 0.001*, > 0.05^a^
*Al* only	9	12.6 ± 1.65	10.3–15.4		37.2 ± 0.4	36.5–37.7	> 0.05*, < 0.01^a^
*Ev* only	11	11.6 ± 2.48	5.0–14.4		37.2 ± 0.9	35.0–38.5	> 0.05*, < 0.01^a^
Ts only	8	13.5 ± 0.77	12.6–14.7		37.2 ± 0.4	36.6–37.9	> 0.05*, < 0.01^a^
No infection	213	12.6 ± 1.66	7.2–16.9	Reference	37.1 ± 0.4*	35.5–38.0	Reference
*Tt* only	1	12.5 ± 0.00	12.5–12.5	ND	37.5 ± 0.0	37.5–37.5	ND
Hw only	1	10.4 ± 0.00	10.4–10.4	ND	37.8 ± 0.0	37.8–37.8	ND
*Pf/Al/Tt*	1	9.6 ± 0.00	9.6–9.6	ND	36.1 ± 0.0	36.1–36.1	ND
*Pf/Al/*Ts	1	9.9 ± 0.00	9.9–9.9	ND	37.9 ± 0.0	37.9–37.9	ND
Sig. difference		*p <* 0.0001					

a probability significance compared with participants infected with *Plasmodium falciparum* alone

* probability significance compared with non-infected participants

ND: Not determined because counts were exactly one.

We used axillary body temperature > 37.5°C to define high temperatures related to pyrexia in the participants at the time of consultation. Patients infected with *Plasmodium falciparum* alone were febrile and had very high significant (*p* < 0.001) mean temperatures (37.9 ± 0.6°C) than participants that were not infected at all (37.1 ± 0.4°C). It was also observed that malaria patients that had single or double helminth coinfections excluding co-infection with *Ascaris/Trichuris* were more febrile and had elevated mean temperatures than patients infected with malaria parasite alone but the difference did not reach statistical significance (*p* > 0.05^a^) Table 4. In addition, patients infected with intestinal helminths alone were apyretic excluding hookworms and had comparable (*p >* 0.05) mean temperatures with non-infected participants but not with their febrile counterparts co-infected with *Plasmodium falciparum* as shown in Table 4. Equally, we observed overlapping distribution of anaemia and pyrexia with malaria in the population (S1 Fig).

### The risk of intestinal helminths on anaemia and pyrexia associated with malaria infection

The prevalence of anaemia was significantly associated with *Plasmodium falciparum* infection (OR = 5.553, 95%CI: 3.241–9.514, *p =* 0.000) than in non-infected participants. However, we observed that malaria patients co-infected with *Ascaris lumbricoides* (OR = 13.712, 95%C.I: 2.552–73.666, *p* = 0.002) and *Enterobius vermicularis* (OR = 16.969, 95%C.I: 1.711–168.265, *p* = 0.014) had significantly increased risk of being anaemic than patients not co-infected. It was also noticed that, malaria patients co-infected with *Taenia* species seem to be susceptible to anaemia than patients that were not co-infected but the difference was not significant [OR = 5.656, 95%C.I: 0.769–41.614, *p =* 0.117]. In addition, patients infected with *Ascaris lumbricoides* or *Enterobius vermicularis* were not susceptible (OR < 1, *p* > 0.05) to anaemia (Table 5). Furthermore, we noticed that patients that were co-infected with *Plasmodium falciparum* and hookworms were all anaemic but the statistics was redundant.

**Table 5:** Association of *Plasmodium falciparum*/Helminths co-infection with anaemia and pyrexia.

Helminths species	Outcome: Anaemia			Outcome: Pyrexia		
	Odd Ratio	95% C.I	*p—*value	Odd Ratio	95% C.I	*p—*value
*Plasmodium* only	5.553	3.241–9.514	0.000	23.630	12.684–44.023	0.000
*Ascaris*	0.356	0.044–2.890	0.457	0.577	0.118–2.821	0.723
*Trichuris*	ND	ND	ND	ND	ND	ND
Hookworms	ND	ND	ND	ND	ND	ND
*Enterobius*	0.608	0.129–2.867	0.734	0.743	0.194–2.856	0.758
*Taenia* spp	ND	ND	ND	0.655	0.130–3.297	0.723
*Plasmodium/Ascaris*	13.712	2.552–73.666	0.002	18.07	3.334–98.000	0.001
*Plasmodium*/Hookworm	ND	ND	ND	ND	ND	ND
*Plasmodium/Enterobius*	16.969	1.711–168.265	0.014	22.560	2.259–225.313	0.007
*Plasmodium/Taenia*	5.656	0.769–41.614	0.117	ND	ND	ND

C.I: Confidence interval. ND: Not determined because count is redundant

Pyrexia was significantly associated (OR = 23.630, 95%C.I: 12.684–44.023, *p =* 0.000) with *Plasmodium falciparum* infection alone than in non-infected participants. Co-infection with *Plasmodium/Ascaris* and *Plasmodium/Enterobius* were significantly (OR > 1, *p <* 0.05) associated with pyrexia than in non co-infected patients. We observed that none of the single helminth species was significantly (OR < 1, *p* > 0.05) associated with pyrexia in infected patients as shown in Table 5.

### Plasma IL-1β levels in malaria and intestinal helminth infected and co-infected patients

**Fig 1:**
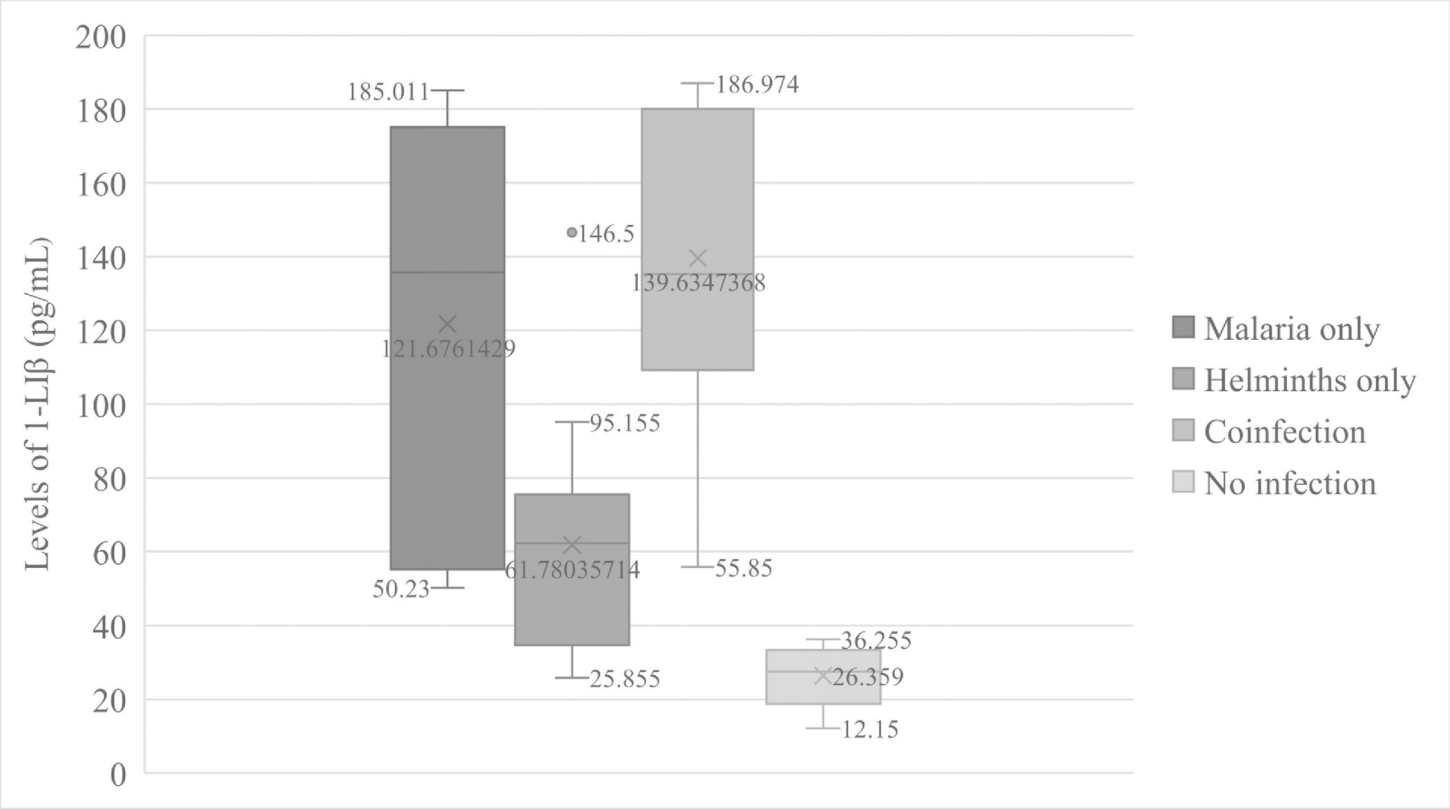
Variations of IL—1β levels according to parasitic disease status.

The overall mean level of IL-1β was 100.03 ± 56.39 pg/mL of plasma. In this study, the results showed an extremely significant effect (F = 18.626, *p =* 0.000) of parasitic infections on the levels of IL-1β in human plasma. We observed that patients infected with *Plasmodium falciparum* alone had significantly (*p* = 0.001) high mean levels of IL-1β (121.68 ± 58.86pg/mL) than participants that were not infected with any parasite (26.36 ± 8.94pg/mL) (Fig 1). At the same time, malaria patients co-infected with intestinal helminths recorded elevated (139.63 ± 38.33pg/mL) mean levels of IL-1β than patients infected with *Plasmodium falciparum* alone (Fig 1) but the difference did not reach statistical significance (*p* = 0.710). In addition, patients that were infected with intestinal helminths alone had significantly (*p* = 0.000) low mean level of plasma IL-1β (61.78 ± 31.69) compared to that of malaria patients co-infected with intestinal helminths. This level was higher than that of participants not infected with any parasite though no statistical significance (*p =* 0.293) was attained. The mean level of the cytokine in patients infected with *Plasmodium falciparum* alone was significantly (*p =* 0.008) higher than the mean level in patients infected with helminths alone as shown in Fig 1.

### Relationship between human IL-1β levels and parasite density

In this study, we noticed that increase in the mean levels of human IL-1β were proportional to increase in malaria parasitaemia. Malaria patients co-infected with single species of intestinal helminths recorded the highest mean levels of human IL-1β pg/mL that significantly (*p =* 0.036) corresponded with the increase in mean malaria parasite density (MMPD) compared to patients infected with *Plasmodium* alone. Malaria patients co-infected with double species of intestinal helminths had lower parasitaemia but with increased mean levels of human IL-1β than in malaria patients alone and in some patients co-infected with single species of intestinal helminths. Lower mean levels of IL-1β were observed in patients infected with intestinal helminths alone as shown in Table 6.

**Table 6:** Mean levels of IL-1β based on parasite densities.

Parasite status	Cases	MMPD ± SD†	MED± SD‡	IL-1β level (pg/mL)
*Pf* only	7	3.41 ± 0.59	-	121.676 ± 56.864
*Pf /Al*	7	3.14 ± 0.69	2.13 ± 0.49	134.726 ± 47.259
*Pf /Hw*	2	3.54 ± 0.34	2.25 ± 0.12	166.375 ± 19.622
*Pf /Ev*	4	3.74 ± 0.19	1.90 ± 0.41	129.409 ± 40.192
*Pf* /Ts	4	3.83 ± 0.27	1.86 ± 0.26	135.799 ± 33.607
*Pf/Al/Tt*	1	2.72 ± 0.00	*Al*: 1.68 ± 0.00 *Tt*: 1.38 ± 0.00	129.425 ± 0.000
*Pf/Al/Ts*	1	2.89 ± 0.00	*Al*: 1.86 ± 0.00 *Ts*: 1.38 ± 0.00	186.974 ± 0.000
*Al* only	4	-	2.25 ± 0.30	55.603 ± 18.688
Ts only	4	-	1.69 ± 0.23	54.070 ±19.689
*Tt* only	1	-	1.85 ± 0.00	65.590 ± 0.000
Hw only	1	-	1.98 ± 0.00	146.500 ± 0.000
*Ev* only	4	-	2.62 ± 0.16	53.536 ± 31.552
No infection	5	-	-	26.359 ± 8.938
Total	45	-	-	-
*p–*value		0.001	0.192	0.036

MMPD: Mean malaria parasite density (parasitaemia)

MED: Mean egg density

### IL-1β levels in anaemic and pyretic patients

**Fig 2:**
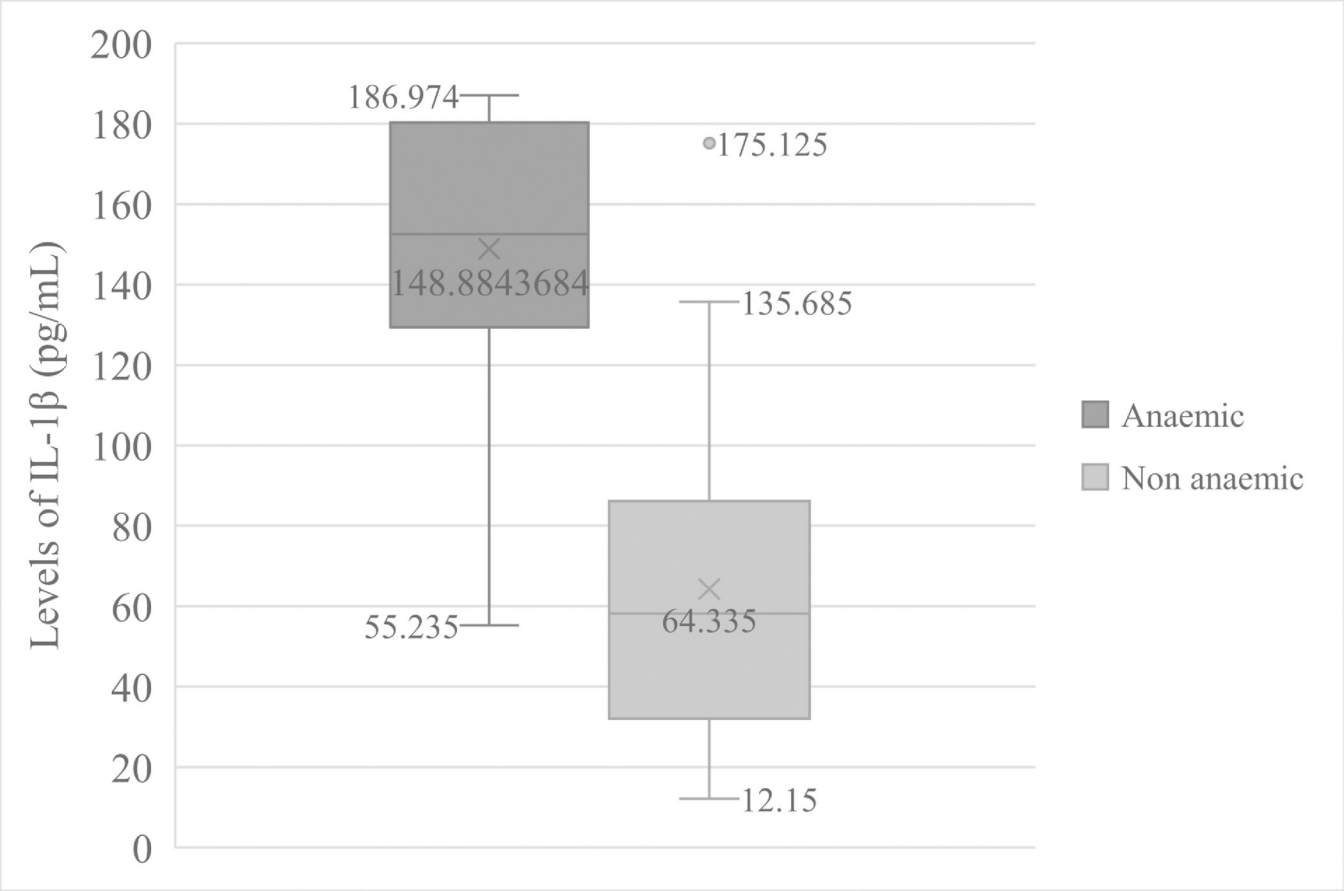
Comparison of IL—1β Levels in anaemic and non-anaemic patients.

**Fig 3:**
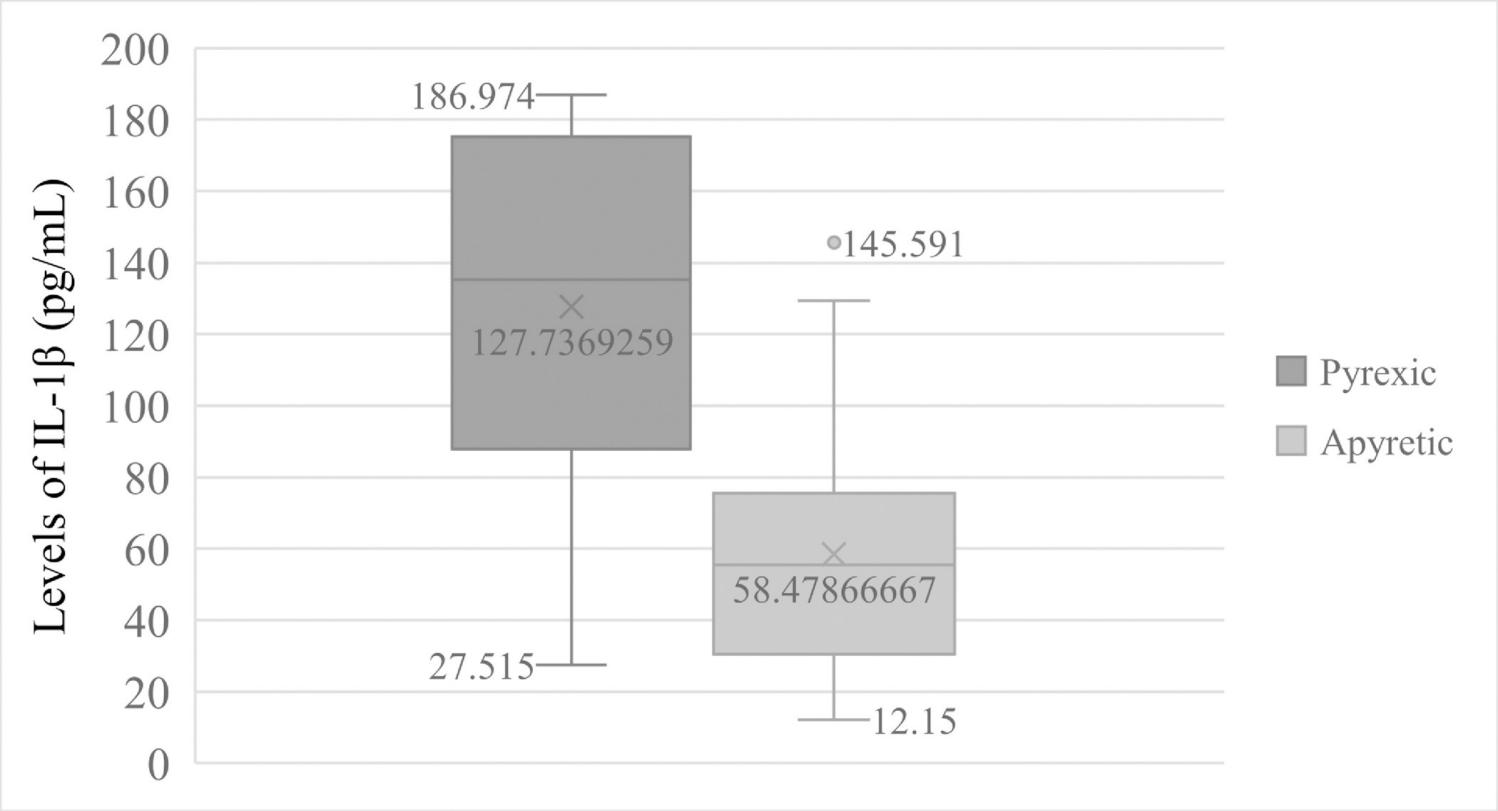
Comparison of IL—1β Levels in pyretic and apyretic patients.

Patients that were anaemic (Hb < 11.0 g/dL of blood) had significantly (t = 7.411, *p =* 0.000) high mean levels of IL-1β (148.884 ± 36.073pg/mL) than non-anaemic participants (64.335 ± 38.995pg/mL) Fig 2. Equally, pyretic patients (Temperature > 37.5°C) also had significant (t = 5.028, *p =* 0.000) higher mean levels of IL-1β (127.737 ± 50.322pg/mL) than in apyretic patients (58.479 ± 36.194pg/mL) Fig 3.

## Discussion

Intestinal helminths frequently coexist in the tropical and subtropical regions especially in impoverished countries and co-infect malaria patients. The precise mechanism of how intestinal helminths affect malaria severities in co-infected patients is not well elucidated. There are speculations about possible immunological implications.

The overall prevalence of *falciparum* malaria in the study population was 30.4%, while that of intestinal helminths was 17.6%. Amongst patients with malaria, 17.4% had intestinal helminths. The most prevalent co-infection was *Plasmodium/Ascaris*. This however recorded lower results compared to some reports in and out of Cameroon [11,41]. *Plasmodium falciparum* and *Ascaris lumbricoides* are highly prolific parasites that are common in tropical areas characterised by poor sanitation and hygiene. This may possibly increase the potential of human coinfection with these parasites and individual lifestyle in such an endemic area is likely to be a risk factor to these diseases and co-infections.

Intestinal helminths were observed to exacerbate malaria parasitaemia in co-infected patients. This is in line with some studies in Cameroon [19,42]. Patients that had co-infection with *Taenia* species and *E*. *vermicularis* registered significantly higher malaria parasitaemia than patients that were not co-infected. More to this, patients that had *Plasmodium/Ascaris* and *Plasmodium/*Hookworm co-infections had heightened malaria parasitaemia than in patients infected with malaria parasite alone but the difference did not reach statistical significant level. Despite non-significant difference of the results, malaria patients that were infected with one or two species of intestinal helminths had a higher risk of coinfection with malaria parasite. This is similar to the work carried out elsewhere in Africa [41]. This increase in malaria parasitaemia in patients infected with different species of intestinal helminths could be attributed to the helminth impact. Intestinal helminths impose nutritional stress on the host that may indirectly reduce the body’s natural resistance to *Plasmodium falciparum*. In addition, helminths modulate immune responses of the host including those against other infecting parasites [26,27] to enhance their establishment. This may obstruct and lessen the normal immune responses of the host that are potent against malaria parasite thus exposing co-infected patients to higher malaria parasitaemia.

The present study revealed a significant impact of helminths on mean haemoglobin concentration levels in patients with malaria. Amongst the helminths, hookworms, *E*. *vermicularis* and *A*. *lumbricoides* were found to be significantly associated with anaemia in malaria patients. This may be due to the fact that adult hookworms cause intestinal blood loss through the ingestion of blood and degradation of haemoglobin. In addition, some reports have associated *E*. *vermicularis* with haemoglobin deficiency [43] and iron deficiency in blood [44]. The establishment and activity of the adult worms in the predilection site of the ileocaecal region of the intestine causes intense inflammations that may lead to ulceration and superficial erosion of the intestine leading to loss of blood. Furthermore, intestinal helminths including *A*. *lumbricoides* cause nutritional stress in the host through food theft that may lead to loss of micronutrients including iron necessary for haemoglobin formation. These may add and exacerbate the anaemia in malaria patients in this study. Intestinal helminth infections alone excluding hookworms were less associated with anaemia. These helminths are mostly luminal and are therefore less associated with intestinal blood loss even though high burdens may contribute to loss of micronutrients but the risk of anaemia is lesser.

Pyrexia is a clinical symptom and a host defence mechanism against some parasitic diseases. Malaria infection is characterised by the production of pyrogens including IL-1β [24] by activated macrophages and from Type-1 (Th-1) and type-2 (Th-2) immune responses. High levels of pyrogens have been associated with pyrexia in patients. In this study, we found out that significantly increased mean levels of IL-1β were associated with pyrexia in malaria patients most especially in patients co-infected with intestinal helminths than in patients infected with helminths alone and participants not infected at all. Independent of the helminth densities, there was no significant difference in the mean temperature of malaria patients co-infected with helminths compared to malaria infection alone. This suggests that helminth infections may indirectly affect the temperature in malaria patients. The modulatory effect of helminths is through the suppression of potent immunological factors in the host including pyrogens [20]. This study demonstrate that intestinal helminths may not have suppressed the levels of IL-1β, and in a cumulative increase, this pyrogen exacerbated the pyrexia in co-infected patients.

In addition, high levels of IL-1β were associated with anaemia and high parasitaemia in this study. Malaria patients co-infected with intestinal helminths showed low haemoglobin concentration and elevated levels of IL-1 β. This demonstrates that intestinal helminths could have increased the production of IL-1β and subsequently caused anaemia in malaria patients. Added to this, we noticed that the high malaria parasitaemia in patients co-infected with intestinal helminths corresponded with increased IL-1β levels even though an insignificantly lower parasitaemia was observed in malaria patients coinfected with *A*. *lumbricoides* and double (*Ascaris/Taenia* and *Ascaris/Trichuris*) species of intestinal helminths. Interleukin-one is associated with cellular immune responses, but in high levels this cytokine result to tissue damage [45]. Thus, increasing levels of IL-1β could cause intestinal helminths to compromise the host’s immune responses through increased production of IL-1β. This may expose malaria patients co-infected with intestinal helminths to higher malaria parasitaemia.

## Conclusions

This study has established that malaria and intestinal helminthiasis co-infect patients in Bamenda. Based on trends in this study, we suggested that the presence of intestinal helminths in malaria patients had deleterious effect on anaemia, pyrexia and exacerbates malaria parasitaemia. In addition, patients infected with intestinal helminths seem to had increased risk of malaria infection and elevated plasma levels of IL-1β were significantly observed in patients with higher parasitaemia, anaemia and pyrexia. This may be due to induced changes in immune activities of the host through increasing IL-1β levels. Further studies are therefore needed for better understanding of changes in immune responses caused by helminths that results to clinical outcomes of malaria and other severities.

Despite some success rate in this study, we observed some limitations that would have broaden and strengthen our study hypotheses. Though this was an observational study, we recorded low counts in patients infected and coinfected with intestinal helminths to ascertain efficient statistical comparison of clinical parameters in these groups of patients. In addition, we understood that demographic characteristics could influence the trends of malaria clinical outcomes in patients with different helminth status. However, no hypothesis to test these factors were formulated during the design of the research protocol. Furthermore, the sensitivity of the diagnostic tool and prevalence of intestinal helminths could be affected by multiple collection of independent stool samples from a single participant, but all of our participants were outpatients on malaria consultation and most of them could only provide single stool or blood samples. This limited our evaluation of the disease severity, assessment of the clinical outcomes and IL-1β levels after receiving treatment protocols from their physicians.

## Supporting information

S1 Text Protocol for ELISA technique used in measuring human plasma IL-1β levels. (DOCX)

S1 Fig The overlapping distribution of anaemia and pyrexia with malaria. (TIF)
