# Supplementary material for: Intestinal helminths as predictors of some malaria clinical outcomes and IL-1β levels in outpatients attending two public hospitals in Bamenda, North West Cameroon
Source: PLoS Negl Trop Dis. 2021 Mar 2;15(3):e0009174. doi: 10.1371/journal.pntd.0009174 (PMC7924769; doi:10.1371/journal.pntd.0009174)
Supplement: S1 Text — (DOCX) [file pntd.0009174.s001.docx]

**Protocol for ELISA technique used in measuring human plasma IL-1β levels**

**Reagents**

| **Component** | **Size / Description** | **Storage / Stability After Preparation** |
| --- | --- | --- |
| IL-1 beta Microplate (Item A) | 96 wells (12 strips x 8 wells) coated with anti Human IL-1 beta. | 1 month at 4°C* |
| Wash Buffer Concentrate (20X) (Item B) | 25 ml of 20X concentrated solution. | 1 month at 4°C |
| Standard Protein (Item C) | 2 vials of Human IL-1 beta. 1 vial is enough to run each standard in duplicate. | 1 week at -80°C |
| Detection Antibody IL-1 beta (Item F) | 2 vials of biotinylated anti-Human IL-1 beta. Each vial is enough to assay half the microplate. | 5 days at 4°C |
| HRP-Streptavidin Concentrate (Item G) | 200 µl 300X concentrated HRP-conjugated streptavidin. | Do not store and reuse. |
| TMB One-Step Substrate Reagent (Item H) | 12 ml of 3,3,5,5'-tetramethylbenzidine (TMB) in buffer solution. | N/A |
| Stop Solution (Item I) | 8 ml of 0.2 M sulfuric acid. | N/A |
| Assay Diluent A (Item D) | 30 ml of diluent buffer, 0.09% sodium azide as preservative. | N/A |
| Assay Diluent B (Item E) | 15 ml of 5X concentrated buffer. | 1 month at 4°C |

**Additional Materials Required**1. Microplate reader capable of measuring absorbance at 450 nm.
2. Precision pipettes to deliver 2 µl to 1 ml volumes.
3. Adjustable 1-25 ml pipettes for reagent preparation.
4. 100 ml and 1 litre graduated cylinders.
5. Absorbent paper.
6. Distilled or deionized water.
7. Log-log graph paper or computer and software for ELISA data analysis.
8. Tubes to prepare standard or Sample dilutions

**Reagent-preparation**1. Bring all reagents and Samples to room temperature (18 - 25ºC) before use.

2. Assay Diluent B (Item E) should be diluted 5-fold with deionized or distilled water before
use.

3. Sample dilution: Assay Diluent A (Item D) should be used for dilution of serum and
plasma Samples. 1X Assay Diluent B (Item E) should be used for dilution of cell culture
supernatant Samples. The suggested dilution for normal serum/plasma is 2 fold.
**Note:** Levels of IL-1 beta may vary between different Samples. Optimal dilution factors
for each Sample must be determined by the investigator.

4. Preparation of standard: Briefly spin a vial of Item C. Add 880 µl Assay Diluent A (for
serum/plasma Samples) or 1X Assay Diluent B (for cell culture medium) into Item C vial
to prepare a 20 ng/ml standard. Dissolve the powder thoroughly by a gentle mix. Add 5
µl IL-1 beta standard from the vial of Item C, into a tube with 995 µl Assay Diluent A or
1X Assay Diluent B to prepare a 100 pg/ml stock standard solution. Pipette 300 µl Assay
Diluent A or 1X Assay Diluent B into each tube. Use the stock standard solution to
produce a dilution series (shown below). Mix each tube thoroughly before the next
transfer. Assay Diluent A or 1X Assay Diluent B serves as the zero standard (0 pg/ml).

| **Std1** | **Std2** | **Std3** | **Std4** | **Std5** | **Std6** | **Std7** | **Zero Standard** |  |  |
| --- | --- | --- | --- | --- | --- | --- | --- | --- | --- |
| **Diluent volume** | Item C+ 880 µl | 995 µl | 300 µl | 300 µl | 300 µl | 300 µl | 300 µl | 300 µl | 300 µl |
| **Conc.** | 20 ng/ml | 100 pg/ml | 40 pg/ml | 16 pg/ml | 6.4 pg/ml | 2.56 pg/ml | 1.02 pg/ml | 0.48 pg/ml | 0 pg/ml |

5. If the Wash Concentrate (20X) (Item B) contains visible crystals, warm to room
temperature and mix gently until dissolved. Dilute 20 ml of Wash Buffer Concentrate into
deionized or distilled water to yield 400 ml of 1X Wash Buffer.

6. Briefly spin the Detection Antibody vial (Item F) before use. Add 100 µl of 1X Assay
Diluent B (Item E) into the vial to prepare a detection antibody concentrate. Pipette up
and down to mix gently (the concentrate can be stored at 4ºC for 5 days). The detection
antibody concentrate should be diluted 80-fold with 1X Assay Diluent B (Item E) and
used in step 5 of Part VI Assay Procedure.

7. Briefly spin the HRP-Streptavidin concentrate vial (Item G) and pipette up and down to
mix gently before use, as precipitates may form during storage. HRP-Streptavidin concentrate should be diluted 300-fold with 1X Assay Diluent B (Item E).
For example: Briefly spin the vial (Item G) and pipette up and down to mix gently. Add
50 µl of HRP-Streptavidin concentrate into a tube with 15 ml 1X Assay Diluent B to
prepare a final 300 fold diluted HRP-Streptavidin solution (don't store the diluted solution
for next day use). Mix well.

**Assay-procedure**1. Bring all reagents and Samples to room temperature (18 - 25ºC) before use. It is
recommended that all standards and Samples be run at least in duplicate.

2. Label removable 8-well strips as appropriate for your experiment.

3. Add 100 µl of each standard (see Reagent Preparation step 3) and Sample into
appropriate wells. Cover wells and incubate for 2.5 hours at room temperature with
gentle-shaking.

4. Discard the solution and wash 4 times with 1X Wash Solution. Wash by filling each well
with Wash Buffer (300 µl) using a multi-channel Pipette or auto washer. Complete
removal of liquid at each step is essential to good performance. After the last wash,
remove any remaining Wash Buffer by aspirating or decanting. Invert the plate and blot
it against clean paper towels.

5. Add 100 µl of 1X prepared biotinylated antibody (Reagent Preparation step 6) to each
well. Incubate for 1 hour at room temperature with gentle shaking.

6. Discard the solution. Repeat the wash as in step 4.

7. Add 100 µl of prepared Streptavidin solution (see Reagent Preparation step 7) to each
well. Incubate for 45 minutes at room temperature with gentle shaking.

8. Discard the solution. Repeat the wash as in step 4.

9. Add 100 µl of TMB One-Step Substrate Reagent (Item H) to each well. Incubate for 30
minutes at room temperature in the dark with gentle shaking.
10. Add 50 µl of Stop Solution (Item I) to each well. Read at 450 nm immediately.
