# Supplementary figures and images for: Intestinal helminths as predictors of some malaria clinical outcomes and IL-1β levels in outpatients attending two public hospitals in Bamenda, North West Cameroon
Source: PLoS Negl Trop Dis. 2021 Mar 2;15(3):e0009174. doi: 10.1371/journal.pntd.0009174 (PMC7924769; doi:10.1371/journal.pntd.0009174)

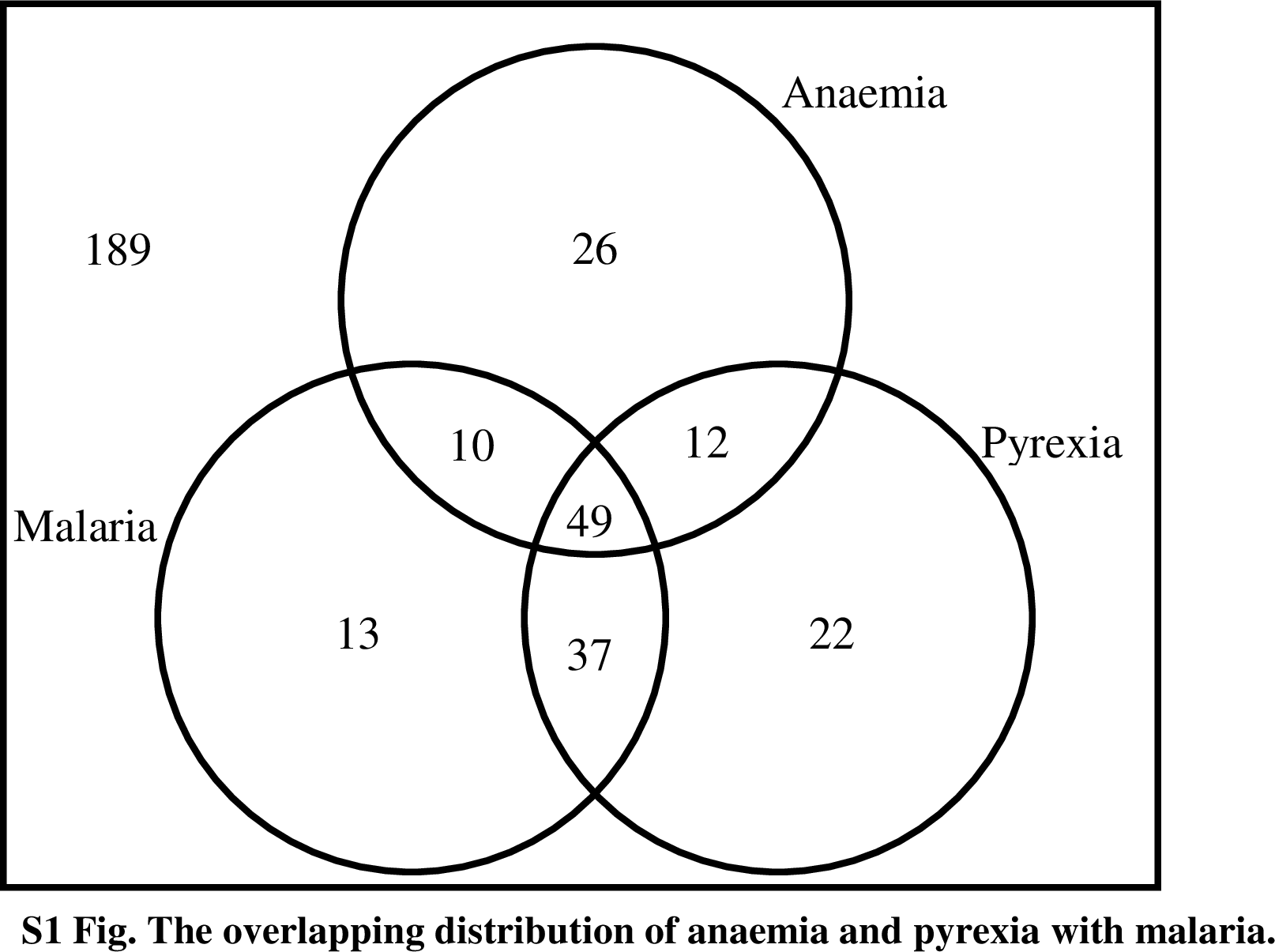

Supplement: S1 Fig — (TIF) [file pntd.0009174.s002.tif]
